# Genetic Association of Multiple Sclerosis with the Marker rs391745 near the Endogenous Retroviral Locus HERV-Fc1: Analysis of Disease Subtypes

**DOI:** 10.1371/journal.pone.0026438

**Published:** 2011-10-25

**Authors:** Bettina Hansen, Annette B. Oturai, Hanne F. Harbo, Elisabeth G. Celius, Kari K. Nissen, Magdalena J. Laska, Helle B. Søndergaard, Thor Petersen, Bjørn A. Nexø

**Affiliations:** 1 Department of Biomedicine, Aarhus University, Aarhus C, Denmark; 2 Danish Multiple Sclerosis Center, Department of Neurology, Copenhagen University Hospital Rigshospitalet, Copenhagen, Denmark; 3 Department of Neurology, Oslo University Hospital, Oslo, Norway; 4 Institute of Clinical Medicine, University of Oslo, Oslo, Norway; 5 Neurology, Aarhus University Hospital, Aarhus C, Denmark; Harvard Medical School, United States of America

## Abstract

We have previously described the occurrence of multiple sclerosis (MS) to be associated with human endogenous retroviruses, specifically the X-linked viral locus HERV-Fc1. The aim of this study was to investigate a possible association of the HERV-Fc1 locus with subtypes of MS. MS patients are generally subdivided into three categories: Remitting/Relapsing and Secondary Progressive, which together constitute Bout Onset MS, and Primary Progressive. In this study of 1181 MS patients and 1886 controls we found that Bout Onset MS was associated with the C-allele of the marker rs391745 near the HERV-Fc1 locus (p = 0.003), while primary progressive disease was not. The ability to see genetic differences between subtypes of MS near this gene speaks for the involvement of the virus HERV-Fc1 locus in modifying the disease course of MS.

## Introduction

Multiple sclerosis (MS) is a chronic inflammatory, demyelinating disease of the central nervous system probably caused by interaction of multiple genes and environmental factors [1]. HLA genes are the most strongly associated genes in MS [2], and low Vitamin D level in serum, Epstein Bar Virus infection and smoking are currently the best documented environmental risk factors [3], [4].

An involvement of retroviruses has also long been suspected [5]. Originally, this was based on animal models, where other demyelinating diseases could be induced by retroviruses [6], [7], [8]. However, these animal diseases are all contagious and horizontally transmitted, and MS does not seem to be transferred in this manner. Therefore, some scientists have focused their research on endogenous retroviruses that are vertically transmitted from parents to offspring as part of the chromosomes. Attempts at isolating such viruses from patients with MS have been partial successful [9], [10]. The main difficulty of the latter studies, apart from the technical challenges, is the inadequacy of Koch's postulates for dealing with ubiquitous agents. Koch's postulates are the criteria traditionally required to be fulfilled in order to establish a causal relationship between a microbe and a disease, and contain the implicit assumption that most people are free of the microbe. The postulates fail, when the microbe is universally present, which is the case with most endogenous viral loci.

Recently, we described the occurrence of MS to be associated with human endogenous retroviruses [11]. The interpretation of these findings did not involve Koch's postulates, but depended on the theories of genetics. We found association of MS with alleles of the host gene *TRIM5* that restricts the replication of a broad variety of retroviruses. This suggested retroviral replication to be involved in this disease, but did not indicate which virus. Hence, we also looked for association of specific endogenous retroviruses with MS. We found an association of MS with markers in and near the endogenous retroviral locus HERV-Fc1, located on the X-chromosome [12]. The associations could be reproduced in several cohorts, encompassing a total of 1060 cases and 2080 controls [11]. The results suggested the involvement of HERV-Fc1 in MS. In addition, we have recently found that expression of HERV-Fc1 RNA is approximately 4-fold higher in plasma from MS patients with recent attacks, compared to plasma from patients in stable remission and from controls [13]. This provides a possible new biomarker and may give clues to further studies of the pathogenic process in MS.

The association of the HERV-Fc1 locus with the clinical course of MS has not been studied previously. MS patients are clinically subdivided into three categories: Remitting/Relapsing MS (RRMS), Secondary Progressive MS (SPMS), and Primary Progressive MS (PPMS). RRMS and SPMS are generally considered time-wise progressions of the same disease, altogether called Bout Onset MS (BOMS) representing the vast majority of the patients (85–90%), while PPMS is characterized by a separate disease entity with disease progression from onset (10–15%). In this paper, encompassing 1181 MS cases and 1886 controls from one new (Norwegian) and one previously examined cohort (Danish), we report that BOMS is associated with a marker near the HERV-Fc1 locus, while PPMS disease is not.

## Materials and Methods

### Ethical approval

Collection of blood samples from MS patients and blood donors for genetic investigations was approved by the Regional Ethics committee (Norway) and local Science Ethical Committees (Denmark). The patients gave written informed consent.

### Clinical data

The sub-typing of the patients was based on clinical data using the criteria of Poser [14].

### Analysis

DNAs were extracted from the blood by conventional means. The DNAs were genotyped for the rs391745 single nucleotide polymorphism (SNP) located near the X-linked viral locus HERV-Fc1 on a mass spectrometry Sequenom® facility using Gold plex reactions (Sequenom, San Diego, CA). The analysis was performed as previously described [11], using the following sequenom primers; rs391745_PCR1: ACGTTGGATGTAAAATATGTGCCCACCCTC; rs391745_PCR2: ACGTTGGATGGATTCTCAGCATGGACCATC and the rs391745 probe ggtgCAGCATGGACCATCTCTTCAG.

### Statistics

The data were analyzed in IBM SPSS version 19 and Excel 2007 (Microsoft, Redmond, WA). For a minority of persons, no subtype or gender information was available, or the typing of rs391745 failed. The main statistic tests performed were χ^2^ tests. To combine p-values we used Fisher's method: χ^2^ (P, 2n) = −2Σ ln (p_i_), where P is the combined two-sided p-value and p_i_ are the n one-sided p-values to be combined.

## Results and Discussion

We analyzed two cohorts of MS patients: 544 cases and 1142 controls from Denmark, and 637 cases and 744 controls from Norway. The Danish cohort was also included in a previous study [11]. Information about individual subtypes of MS had been collected for most patients in both cohorts.

In the previous analysis, the best marker in or near the HERV-Fc1 locus for association with MS was rs391745. Table 1 shows the association of MS and subtypes of MS with rs391745 in the two cohorts. Both cohorts showed an association of the C-allele of the rs391745 SNP in the total MS group (p = 0.003). The overrepresentation of C-alleles among cases corresponds to what was observed previously and extends previous findings to a larger set of patients. BOMS also showed a significant association to the C-allele of this SNP (p = 0.003). In contrast, PPMS was not associated with any allele of rs391745. This lack of association was present in both cohorts and was emphasized when the cohorts were combined.

**Table 1:** Association of rs391745 located near the HERV-FC1 locus and Multiple Sclerosis.

Cohort	Status	C-allele carriers n = 467	C-allele noncarriers n = 2531	Frequency of C-allelecarriers	OR^1^ (CI95%)^2^ (2-sided)	P-value (2-sided)
Danish	All cases	102	431	0.19	1.35(1.03–1.75)	0.03
	PPMS	4	29	0.12	0.79 (0.27–2.26)	0.7
	BOMS	86	359	0.19	1.36 (1.02–1.81))	0.03
	Controls	166	945	0.15		
Norwegian	All cases	105	522	0.17	1.35 (1.00–1.83)	0.05
	PPMS	16	91	0.15	1.18 (0.67–2.10)	0.6
	BOMS	85	411	0.17	1.39 (1.01–1.92)	0.04
	Controls	94	633	0.13		
Combined	All cases	207	953	0.18	1.32 (1.08–1.61)	0.003
	PPMS	20	120	0.14	1.01 (0.62–1.6)	0.96
	BOMS	171	770	0.18	1.35 (1.09–1.67)	0.003
	Controls	260	1578	0.14		

Of the persons 70 failed determination of rs391745, and 59 lacked information of MS subtype.

1 OR: oddsratio for C-allele carriers vs C-allele noncarriers.

2 CI95%: 95 percent confidence interva.

To make sure gender issues did not perturb the results, we also performed a gender-stratified analysis of the combined cohorts. The conclusions were the same: BOMS was associated with HERV-Fc1, PPMS was not (Table S1). The p-values were higher, but this was to be expected as the stratified analysis does not contain any cross comparisons: male cases versus female controls and vice versa and thus has less strength.

Three arguments strengthen the tie between MS and HERV-Fc1. First, the association of MS with markers on the X-chromosomal region is limited to an approximately 20 kb region, which includes HERV-Fc1. There are no other known genes in this region [11]. Secondly, the restriction gene *TRIM5*, which is known to limit retroviral replication, influences the risk of getting MS [11]. Finally, we have found that expression of HERV-Fc1 RNA is four-fold increased in the plasma of MS patients with a recent history of an attack, relative to patients in stable remission and to controls [13]. We take all these data as indications that development of BOMS may be influenced by the endogenous retrovirus locus HERV-Fc1.

Another corroboration to this conclusion stems from the observation that PPMS has a gender-ratio close to one [15]. This is in contrast to the near 2∶1 ratio observed for BOMS. This difference in gender ratio was to be expected if HERV-Fc1, located on the X-chromosome, plays a role in BOMS but not in PPMS.

It seems reasonable to speculate on the importance of HERV-Fc1 for disease. If we take the epidemiological approach and assume that rs391745 is the causative variation, we arrive at an etiological fraction of 0.05 to 0.1, i.e. rs391745 is a minor risk determinant for BOMS. Alternatively, if we take the genetic view that rs391745 is a marker in linkage disequilibrium with the true causative variation, the etiological fraction of the latter variation should be higher and the virus could in principle be a primary cause of BOMS. With the latter optimistic view in mind, we are searching for markers with better association to BOMS in and around HERV-Fc1.

Recently, a large genome-wide association study (GWAS) of MS identified an excess of fifty different genes statistically associated to MS [16]. An overrepresentation was observed of genes related to the immune system. This GWAS did not identify association to the HERV-Fc1region, but this is not in conflict with the HERV-Fc1 data presented here.

In conclusion, this study suggests a molecular distinction of different disease courses in MS by involvement of HERV-Fc1. This warrants further investigations.

## Supporting Information

Table S1 **Gender-specific p-values and odds-ratios for rs391745 in relation to subtypes of MS.**
(DOC)
