# Supplementary material for: Genetic Association of Multiple Sclerosis with the Marker rs391745 near the Endogenous Retroviral Locus HERV-Fc1: Analysis of Disease Subtypes
Source: PLoS One. 2011 Oct 25;6(10):e26438. doi: 10.1371/journal.pone.0026438 (PMC3201946; doi:10.1371/journal.pone.0026438)
Supplement: Table S1 — Gender-specific p-values and odds-ratios for rs391745 in relation to subtypes of MS. (DOC) [file pone.0026438.s001.doc]

Table S1. Gender-specific p-values and odds-ratios for rs391745 in relation to subtypes of MS.

| Gender | Disease subtype | C-allele carriers | C-allele noncarriers | OR^1,3^ (CI95%)^2^ (2-sided) | P-value (2-sided) |
| --- | --- | --- | --- | --- | --- |
| Male | All | 43 | 306 | 1.51(1.00 -2.30) | 0.05 |
|  | PPMS | 8 | 53 | 1.63 (0.74 – 3.58) | 0.22 |
|  | BOMS | 32 | 233 | 1.48 (0.94 – 2.34) | 0.09 |
|  | Controls | 60 | 648 |  |  |
| Female | All | 162 | 646 | 1.16 (0.92 – 1.46) | 0.21 |
|  | PPMS | 12 | 67 | 0.83 (0.44 – 1.56) | 0.63 |
|  | BOMS | 139 | 537 | 1.20 (0.94 – 1.52) | 0.14 |
|  | Controls | 200 | 925 |  |  |
| Combined | All |  |  |  | 0.018^4^ |
|  | PPMS |  |  |  | 0.64^4^ |
|  | BOMS |  |  |  | 0.021^4^ |
|  | Controls |  |  |  |  |

Of the persons 13 lacked information about gender.

1. Oddsratio
2. 95 percent confidence interval
3. OR for males C vs G; OR for females CC and CG vs GG, ie. C-carriers vs non C-carriers.
4. p-values for the combined genders were calculated from the p-values of the single genders using Fisher’s method for the combination of p-values.
